# Personal

**Published:** 1890-12

**Authors:** 


### Personal.

Dr. I. S. Stone, formerly of Lincoln, Virginia, and Surgeon in charge of the Springdale Sanitarium, has removed to 1309 H street northwest, Washington, D. C., where he will devote his entire time to that line of work in which he has been so successful — gynecology. Dr. Stone is one of the most energetic and progressive physicians in Virginia. He has always been an active Fellow of his State Society, and more recently served well the State Board Medical Examiners as a representative of the State at large. Success must follow him wherever he goes.— *Virginia Med. Monthly.*

---

Dr. Seneca D. Powell, of New York, gave a dinner at his residence, No. 12 W. Fortieth street, on Wednesday evening, November 12, 1890, in honor of Dr. William H. Johnston, of Birmingham, Ala. The occasion was a delightful one in all respects, and it afforded the distinguished guest an opportunity to meet and renew acquaintance with his many friends in New York, where in former years he resided, and who were delighted to revive the memories of younger days under such pleasant auspices.

